# London School of Dental Surgery.—Mr. Cartwright's Inaugural Address

**Published:** 1860-07

**Authors:** 


### SELECTED ARTICLES.

### ARTICLE VIII.

### *London School of Dental Surgery.*—Mr. Cartwright's *Inaugural Address.*

Gentlemen,—On an occasion like the present, which presents an important era in the annals of Dental Surgery, inasmuch as this is the inauguration of a new school of instruction in our special department of practice, it has been thought fitting that an introductory address should be delivered, and the honor of giving that address has been deputed to me. I acknowledge the honor, and I feel a pleasure in the task; but I own at the same time to being possessed of misgivings lest the discourse I am about to submit to you shall not realise your anticipations. Considering the especiel nature of the occasion, I have departed from the usual character of introductory lectures; and, instead of making this the herald of what is to come, and entering into the history of the subject of the different lectures about to be given on Dental Anatomy and Physiology, Surgery, Mechanics, and Metallurgy, I have contented myself with the endeavor to show you, who represent to a certain extent a casaul audience here to night, what the present state of Dental Surgery (and I may say Dental Politics) is, and what we trust it will be, in a few years' time, when the measures which have been lately carried out at the College of Surgeons, for encouraging the better and more uniform education of dental practitioners, have come into full play.

It is with feelings, too, of pleasure and no little pride that I find myself occupying the post of lecturer in a school formed on so sound and satisfactory a basis, and the first educational institution for dental purposes ever proposed to be established in this kingdom. When I tendered my services to the committee, I was not insensible of the responsibility I incurred ; and, occupied as my time is in the duties of private practice, I should have shrunk from undertaking the extra labor involved in carrying on the duties attached to the office I hold in the hospital and teacher in the school, had I not felt that I should be acting in opposition to my professions of interest felt in regard to this school, and the part I have taken from the first in assisting in its formation and the carrying out of the various stages of the scheme. And, again, I could not but feel that, as the son of a man who, with so much ability and credit to himself, maintained for so long the foremost rank in the profession, and by his great and universally-recognized talents and strict professional rectitude did so much towards the elevation and improvement of dental practice, I should not be doing right if I refused from personal considerations to come forward and assist with my best endeavors a work so unselfishly commenced, so zealously and judiciously carried on, in spite of obstacles and opposition, now so satisfactorily brought to a conclusion, and so cheeringly yielding a return for all the labor and trouble which has been bestowed. I cannot help feeling that I have undertaken a difficult task, and one which causes me some anxiety ; for I must be subject to the criticism of my professional brethren, to whose approbation I naturally look with solicitude ; for the possession of their good opinion I hold to be the earnest of character and position.

If we compare the state of our profession at the present time with what it was at the beginning of the present century, we must be struck with the great change which has taken place in it. Not only has the number of practitioners enormously increased, but the quality of work done, both in the surgical and mechanical departments, has generally improved in a very satisfactory degree; and where a few bright ornaments existed who upheld by their successful operations the credit of British Dental Surgery, there exist now many whose operative excellence and mechanical ingenuity are highly creditable to them as individuals, and who as a body, conduce to the elevation of English Dental practice at home and abroad. It must be remembered, too, that this general advance has taken place under circumstanccs of disadvantage, and without any recognized system of education. Individual labor has overcome difficulties; but the present state of perfection of operations has, with some exception, been acquired by experience gained at a time when a knowledge of the principles of such operations, and the way to perform them, ought to have been acquired before; in other words, the dentist has been *in statu populari* at the time when he ought to have been a proficient practitioner, the first years of practice being spent in learning the operative branch of his calling, and often feeling deeply his inability to do conscientious justice to those who placed themselves under his charge. Such has been the inevitable position of many who commenced practice—even those who, in other respects, were, theoretically, generally well educated and qualified by mechanical aptitude acquired in the work-room, either as articled pupils or otherwise. But there is a large class of persons in practice who can have had no possible opportunity of acquiring the knowledge which is, in this day, imperative for a professional man; and, consequently, many are driven to seek for patients through the medium of advertisements, whilst others, not possessing a particle of knowledge of the duties attached to the calling they profess to follow, are utterly lost to all sense of moral rectitude and self-respect, obtain a migratory practice by means of falsehood and exaggeration put forth with unblushing impudence.

In a profession which has existed for so long a time without governance or control of any kind, and when any one might style himself a surgeon dentist, and practice without let or hindrance, ability or qualification, discord and misunderstanding can hardly fail to arise when an effort is made to raise the standard of professional acquirement. Many will dread the idea of education; some will choose to think that the exertions of those who are endeavoring to raise their profession emanate from selfishness, and will strive to misrepresent the motives which dictated such exertions; but it must be remembered that to do a good action, and to fight in a new cause, require no small amount of patience and determination on the part of those who undertake the duty, and it is seldom that the immediate results compensate the time and trouble which have been expended. If the action, however, be really good, and the cause fought for good, sound, and noble, difficulties will be surmounted, and the struggle end in victory.

A feeling has long been prominent in the minds of the majority of intelligent practitioners, that an educational system was urgently called for, in order that the profession of the dentist should become a recognized profession; recognized by its intelligence, and commanding respect and position by the intellectual acquirements of its members. In furtherance of the fulfilment of this long-cherished desire, for some time past (upwards of four years,) a number of gentlemen have devoted much of their time and talents in originating and maturing a scheme of education which will advance the present standing of the dentist, and must prove of very essential service to the rising aspirants to dental practice. Although the circumstances connected with the details and carrying through the various stages of the scheme have necessarily entailed delay in the opening of the hospital, and especially the school, yet it is a great satisfaction to those who have so earnestly labored, and those who have shown an interest in the measures proposed, to know that the idea of a school, propounded on broad views of instruction in dental science, emanated from those who have all along been desirous to the dentists as a body recognized by, and associated with, the College of Surgeons ; aud though another school has been the first in the field, it must not be forgotten that the curriculum presented there, is in most respects an imitation of the scheme proposed here long ago.

But, whatever differnces may exist as to the policy of the College of Dentists in regard to the diploma, no one connected with the school, or who has a part in giving it vitality, wishes to cavil at or oppose its educational movement, since the formation of such institutions, if ably conducted, must conduce to the common weal.

Judging from the introductory remarks which have been made by different lecturers at that school, the idea of the required curriculum is in most respects identical with our own ; and where a difference appears to exist, it is the result of misunderstanding or misrepresentation. It is foreign to the object of this discourse to dwell upon differences which unfortunately exist as to the proper place from whence a charter or diploma should emanate ; but, regretting as I do, and I am sure all do, who have fairly considered the subject, or taken an active part in the question, the misunderstanding which is rife, I cannot avoid touching on the subject, as I deem it but just and proper that the relative views and acts of the two educational bodies should be clearly represented and understood.

The object of those who form a numerous and respectable portion of the dental profession, and who met together before the College of Dentists was conceived, was to connect, if possible, the dentists as a body with the College of Surgeons ; if that scheme proved not feasible, then to create an institution and to seek a charter for themselves, which would empower them to grant diplomas in dental surgery. From these views those who were entrusted with the conduct of affairs never turned aside, never were in haste to forestall other measures by premature movements, until they were placed in a position which would enable them to carry out their schemes without having to retrace their steps. They waited patiently until the council of the College of Surgeons had well considered their memorial, and either sanctioned or refused their request. Time has proved the worth of the suggestions contained in that memorial, and the council of the College of Surgeons, after free discussion and inquiry, have secured the charter empowering them to grant special examinations for those who intend to practice as dentists; and, having approved of the curriculum which was subjected for their consideration, have chosen the examiners, who have, as you know, already commenced their duties, and about seventy gentlemen have already obtained the certificate of competency to practice. To-day we witness the first fruits of the labor of the council and members of the Odontological Society—a society which was formed as the rallying point for those who desired to be what is now so satisfactorily realized. We do not profess here to grant degrees which can be of no value; but we hope and will strive to assist in educating to the best of our abilities, and by our example, those who will in the course of time take our places, whenever hurrying time has laid his hand upon us, so as to enable them to start in life with a knowledge of their profession in all its phases, and to hold a position in the medical world, members of an educated and qualified profession.

The College of Dentists was formed, apparently, with the view of detaching the dental profession from the general profession of medicine; and in the first instance, they ignored altogether the idea of connection with the College of Surgeons. Although the ruling powers there have from time to time modified their opinions, they have now published their intention to seek a charter for themselves, (with what success remains to be seen,) and they have formed a school in which the curriculum appears to be more restricted than should be; and the position they would take is certainly not so high as they will take who seek the dental diploma granted at the College of Surgeons. And this appears to be a view in accordance with the opinion of many present and former members of that college, as in the published lists of those who have obtained the new diploma are to be seen the names of several gentlemen who either are or were recently connected with that institution.

That great service has been done for our profession by these measures (I refer to our connection with the College of Surgeons,) needs no demonstration on my part; and, although their full value cannot be experienced for some few years, yet the immediate results will be important, inasmuch as the public will appreciate the movement. And let me say that we are not too early. The measures, indeed, ought to have been carried out long ago. Although we have amongst ourselves practitioners equal in every respect to the best practitioners of any other country, still it must be acknowledged that the dental institutions of America have sent forth a great number of gentlemen who form a body of dentists of a high and uniform standard, and scattered over the world they exhibit a degree of excellence, individually, which is conclusive of the value of their practical and systematic education. All we require now is union, not schism, and a desire to do the best for our common weal, and the dental profession of England as a whole will command respect and position; to effect this we must be true to ourselves, and, by freely discussing questions of difference, and courting inquiry, trust to the sound sense of the majority of our brethren, and there will be little fear for the right views being supported. In this school it is considered essential that the dentist should be acquainted with the principles and practice of surgery, and a thorough knowledge of general anatomy and physiology; that his efficiency in these subjects should be tested by examination undergone at the highest surgical examining board—such a curriculum, indeed, as will enable the possessor of the special dental diploma to seek the membership, and eventually the scholarship of the college, if he choose to do so. There exists too much intelligence and proper pride among us to rest content with a position little short of that of the mechanist; and although it may be put forth to unreflecting minds that a superficial knowledge is all the dentist requires, with apparently some success, still it is an error of so grave a character that such a doctrine can have little root.

There have been of late several introductory discourses, which have fully treated of the history of dental surgery, and which have been published and circulated in the form of pamphlets, and in the pages of dental and medical journals: they generally take a full view of the subject, and therefore I do not deem it necessary to take up your time in reiterating what I have said to you on a former occasion at another institution, or what has been very well said on more recent occasions by others. Mr. Alexander Nasmyth's historical introduction to a work he unfortunately did not live to perfect, contains a very full and careful digest of the writings of most authors, from the earliest times to those of our own. Mr. Ibbetson's introductory lecture given lately at University College, and Mr. Grimshaw's at Queen's College, Dublin, have fully expressed as much as can or need be said on the subject; and in the British and Foreign Quarterly Review is a very good review of the works and opinions of Messrs. Huxley, Rainey, and Tomes; and I may add Magitot's Study on the Structure and Development of the Human Teeth, which contains an ample historical record of the writings of various authors.

From the earliest times Dental Surgery has been considered a part of surgery proper, and the majority of those who have written on the physiology and surgery of the teeth, in the last and preceding century, were eminent surgeons and physiologists of their time. Among the Egyptians, Greeks, and Romans, the conduct of the teeth was practiced as a specialty; in Pompeii, we know, implements have been found which were evidently used in operations on the mouth, and are, indeed, deposited in the museum at Naples. Still, the writings of the old Greek authors make it evident that dental practice was carried on in connection with general surgery. It is in recent times that dental surgery has assumed a really special branch of practice; a fact to be accounted for in the increasing necessity of the aid of the dentist, and consequently on the improved manipulative practice, which requires that more time should be devoted to the operations.

It is gratifying to mark the progress which has taken place recently in all the branches which, combined, constitute dental science. In anatomy and physiology especially, in reference to the minute structure of the teeth, and the phenomena connected with their development and growth, as well as the maxillary bones, much has been done; and it is satisfactory to find that among those who have zealously assisted to bring to light the secret handicraft of nature to unravel the laws which govern absorption and reproduction, are several members of the dental profession. The works of Blake, Fox, Bell, Robertson, Koecker, Nasmyth, and Tomes; Chaplin, Harris, Bond, Arthur, and others of America; Blanden, Serres, Oudet, De la Barre, of France, offer no mean array of professional literature. It is with especial pleasure that I refer to a work which has most recently issued from the press—the "Manual of Dental Surgery," by my colleague, Mr. Tomes; by that work and the lectures which he previously published, and papers read at the meetings of the Royal Society, he has earned a good name, and made for himself a position in his profession which entitles him to the respect of all. Although the mechanical branch of the profession has been much improved in quality of work, and greatly extended, yet no work on that subject has been published in England, as yet, on a comprehensive scale; but I hope we shall soon have such a work, embracing chemistry and metallurgy, as applied to the dentist's art, the manufacture and tempering of steel for instruments—in fact, all the subjects which can be embraced in mechanical dentistry, so that we may have a thoroughly well-got-up book of reference for that particular department. As a body the dental profession has not heretofore taken, nor does it at the present time take, a good position in the community; and it is not a difficult matter, although it may be an unsatisfactory truism which one almost shrinks from confessing, to discover a solution to the fact that dentists are, to use a common phrase, "looked down upon" in society. Up to the present time there has been, it must be confessed, a want of education among the majority—too great a tendency to carry on practice more like a trade than a profession, which naturally leads the public to look on dental practitioners in the light of mechanical handicraftsmen, rather than as professional men possessing scientifie intelligence and educational acquirements. I do not make these remarks with any intention of making invidious or ill-natured reflections. It has been generally felt that the profession does not stand as it should, and that feeling and the knowledge of the cause have urged the adoption of such measures as in time will improve the status and compel society to look with more respect upon the profession and its members. No one who practices as a dentist, and, by his sense of duty and his own regard for his *amour propre* and his calling, so conducts his practice that it may be worthy of the name of a profession, can see day after day the lists of advertisements which appear so prominently in the *Times* and other papers, without a sense of shame and feeling of strong regret; the inducements held out, and the fabulous promises made, failing as they must to realize the expectations and confidence of those who are allured by such professions, naturally bring discredit which, to a great extent, spreads its influence on the profession generally.

It has been objected, when canvassing the importance of a more general and systematic education for dentists, and a connection with the College of Surgeons, that it is useless—that the mechanical department constitutes it a trade, and as a trade it ought not to aspire to a diploma, and that it can have nothing in common with the College of Surgeons, and that special examinations may as well be issued for ophthalmic, orthopædic, or aural surgeons. There is little argument needed to show the fallacy of such reasoning. The eye, the ear, and those diseases and deformities which are commonly embraced in orthopædic surgery, are parts of surgery. The tissues and structures in health and disease are identical with all parts of the system, and the necessary operations and remedial treatment are amenable to the same laws which affect the tissues when diseased, abnormal or injured by accident, in any other part of the body. In large cities many a man of acknowledged ability and general professional acquirement is driven by force of circumstances, and in spite of his endeavors to the contrary, into special practice. There exists in the minds of the public a strong bias for consulting those who have a name as famous in particular lines of practice, and thus one man finds that he is specially consulted for the eye, another for the ear, this for the chest, and that one for the stomach, and so on, almost *ad infinitum*. Dental surgery is a specialty in the strict sense of the word : the operations necessary for the eradication or cure of the diseased structures of the teeth require nice manipulation, and a steadiness of hand, which can be gained only by systematic education of the hand and fingers ; and the loss of teeth can only be supplied by the aid of mechanical ingenuity and aptitude ; but the necessity for mechanical appliance does not remove dental surgery from surgery proper, more than the necessary mehanical appliances remove orthopædic surgery from general surgery. The curative treatment of surgery is in a great measure mechanical, and he makes the best surgeon who possesses, in combination with intellectual capacity, what is termed a mechanical turn of mind. The crooked lim made straight, the curved spine supported or brought again to its normal state, the cicatrices from burns absorbed by extension by mechanical means, the necessity for the knife being abandoned, are the triumphs of mechanical surgery.

That dental practitioners are a useful body of men is indisputable. The number of dentists in the United Kingdom, upwards of 1,500, show that as a community it is important. The mechanical department has nothing connected with it which is degrading, and the preliminary education of the dentist, when properly carried out, as in future it will be, is on a par with medicine or the collateral sciences. Dental science, indeed, is an occupation which gives to the mind taste and feeling for the art generally. An artist's eye, hand, and mind is a necessity indeed; for perfection of workmanship is the aim of the honest practitioner, and besides the securing the comfort of the patient, the expression and character of the mouth must be studied when the dentist is called upon to supply the ravages which time or disease has made. It cannot be maintained with success that a knowledge of medicine is unnecessary, or that a knowledge of anatomy and physiology is not required. Local and remote pains exist, which may either arise from the teeth, or may have origin in disordered conditions of remote organs and deranged functions. Clenching or grinding of the jaws in sleep or otherwise may be the result of cerebral or spinal irritation, as likewise diseases of the chylo-poetic viscera and hemorrhoidal condition of the rectum, whereby is induced, indirectly, considerable tenderness of the teeth and sockets together with irritation or inflammation of the gums. An irritable condition of the mucous membrane lining the stomach may, when excited by undigested food or flatus, cause neuralgic pains of great intensity, passing up the œsophagus and extending along the course of the throat, palate, soft and hard, and dental nerves of the superior but most commonly of the inferior maxilla. Or a tooth, apparently without sensation, may be the cause of pain or tenderness in the region of the articulation of the jaw, wandering, sharp, darting pains in the face, head, temples, or neck, or severe pain in the eye, ear, limbs, or hip, and diseases or the periodical excitement of certain organs, the uterus more particularly, may sympathetically excite pain in the teeth.

Unfortunately for humanity, the teeth appear to be a portion of our frame peculiarly liable to premature decay. Considering their importance in the economy, it seems strange that they should be so deficient in those qualities the perfection of which is essential to the health and comfort of individuals ; in fact, it appears that of all parts of our system the teeth are the least adapted for the office they have to fulfil ; or to take a superficial view of the question, one would be inclined to attribute a departure from the harmonious action so constantly recognisable in the laws which regulate our frame, and balance so nicely the phenomena of life and health. Whatever the cause be which entails frequent disease and loss of the organs of mastication (and it will be my province hereafter to inquire into the causes of such failure,) their prominent importance as the mechanism whereby the animal and vegetable substances taken to supply nutrition, and the diseases and discomfort which ensue when that mechanism is defective or destroyed, give value to the aid of the dentist, and prove that it is essentially conducive to the health of mankind. By the formation of the dental hospital a class of persons who suffer greatly, but who have not the means to command professional aid, have the opportunity afforded them of being relieved ; and this school of instruction in all the branches which are comprised in dental surgery will teach aspirants to that science how to effect all the good that can be done for the relief of suffering, as regards especially the mouth. One great result will be gained by the establishment of these institutions, inasmuch as together they will be the means of obtaining important and valuable statistical facts which may go far to throw light on much that is obscure as relates to the cause of diseases, and the phenomena of many of the affections exhibited in the structure or development of the teeth. In the treatment of disease there is much that is empirical; and here again the system of treatment may become more generalised and systematic. It is by recording facts as they occur that truly valuable facts can be obtained, and hence the importance of keeping careful records of all that is done in the operating room of the hospital. I cannot impress too strongly on the minds of you who are present here to-day as pupils the after value which you will attach to notes of cases systematically registered. On comparing your impression of the results of practice, whether in reference to numbers or cases, you will be surprised, on referring to your notes or tables, to find how erroneous has your mental impression been.

There are points in practice at the present time which are at variance with the experience of older practitioners. These institutions will be the means of elucidating the value of particular lines of practice, and testing the motives which have urged them into prominence. Truthfulness is the basis of all scientific research, and should ever be the household god of the professional man. It requires caution as to the reception of new doctrines, and we must carefully discriminate between a theory hastily promulgated from interested motives, and a fact put forward on philosophic grounds, or one emanating in scientific honesty. If long experience prove that in jaws which are abnormally small a crowded set of teeth may be prevented or remedied by the timely removal of certain teeth, and an individual states that removing teeth under such circumstances is altogether unnecessary, and that the crowded teeth should be forcibly drawn or forced into a circle, truth must rest on the side of one of the modes of practice only; or if dead and painful teeth are retained in the mouth, on the principle that it is bad practice to remove teeth, whilst experience proves that teeth in such conditions are useless members, and worse than useless, inasmuch as they interfere with free mastication, the value of the practice which forbids the removal of such teeth must be substantiated before the practice which has been sanctioned by experience is laid aside. Points of practice, then, such as these, will be tested by results based on extended information conveyed by analyses of facts, and a beneficial influence will be exerted on operative dental surgery.

It has been asserted that in practice dental surgery is gone back, and that bad teeth and alveolar abscess are much more common than was formerly the case, and that the prevalent bad condition of the teeth and gums is owing to the use of amalgam filling. I must say that, as far as my experience goes, the inference is adduced from narrow channels, and I am quite certain of this fact, that amalgam fillings do not give rise to alveolar irritation *per se* Doubtless the endeavor to preserve teeth is now-a-days greater than it was in years gone by; and constantly amalgams are used for convenience, and in cases of extensive decay, for comparative plainlessness and safety as regards fracture of the thin walls of the cavity. Were gold used in all such cases, the result would be the same as regards subsequent alveolar irritation in a certain number of the cases treated. In former years, numerous teeth which are now more or less successfully plugged would have been extracted, and hence arises the fact—if it be a fact—of the greater prevalence of such conditions. But here again it behooves us to look closely into assertions, and convince ourselves of their value. We must consider the number of dentists who exist now as compared to former years, the number in whose practice such chronic disease exists after teeth are filled in but few exceptional cases, and who as practitioners equal, if they do not excel, those who practiced at the beginning of the century; and we must not forget the number who really know but little concerning the treatment of teeth, and whose ideas of filling cavities are limited.

A prominent feature of the present day, and one comparatively of recent date, is the craving for painless operations. Teeth are now expected to be removed without the sensation which normally accompanies the operation ; and the various stages even of filling cavities in teeth are to be gone through without disturbing the nervous sensibility of the patient. The operator who applies the force necessary to consolidate a sheet or more of gold leaf into a solid plug is not unfrequently taxed with being extremely rough. In order to overcome the repugnance or inability of the present race to bear even a moderate degree of pain, agents are used which deaden sensibility or produce anæsthesia ; freezing mixtures and electricity effecting, or being supposed to effect, the one, and chloroform, and we hear of mesmerism—hypnotism more recently—producing the latter. These several agents have, or have had, each their supporters and admirers, and appear to have been successful in various degrees.

Imbued as society is now with the notion that pain is next to unnecessary, efficient operations are rendered more difficult and troublesome, and often become extremely irksome to perform ; unsatisfactory results consequently ensue from the frequent complaining and impatience of the patient, and the sometimes yielding of the better judgment of the operator.

It is the province of this hospital and school to test and record the effects of agents now commonly in use for deadening sensibility or mitigating pain, so that the profession at large may have reliable data to go upon as regards their capability really to effect what is reported of them.

It is natural enough that the public should expect from practitioners the utmost assistance that can be given, and if certain agents will ensure them from suffering they will seek such aid where they can find it ; and it behooves all practitioners to satisfy themselves as to the value of any new agent which may from time to time be introduced. It is, however, equally incumbent upon them sternly to avoid pandering to weakness and folly. If they be reasonably satisfied that any particular line of practice is empirical, no consideration of temporary credit or gain should induce them to have recourse to it. It is the duty of all to be very careful of maintaining a strict professional moral code. There are numbers of unthinking people who may be deceived by self-praise, or easily misled by the profession of superior excellence, however offensively paraded, or may be deceived by an unworthy criticism or unjust remark passed upon the work or ability of a brother professional. But there are many also who are not to be caught by such discreditable conduct, and who form their own just estimate of the individual who adopts such means of winning patients. Let there be honorable competition among professional men. He who attains and maintains a high position by his own labor and ability, need be no object of envy and jealousy. His example should rather be to others the stimulus to exertion, his successful career deemed worthy of applause and imitation.

There is a feature effecting the present state of dental practice which demands a special notice on an occasion like the present; and that is the passing away of certain classes of disease, and certain lines of practice, from the province of the dentist into the hands of surgeons and physicians—a change in some respects natural, in others not altogether to the advantage of the public. As a general rule, those affections which depend upon causes and conditions which constantly present themselves to the notice and consideration of special classes of practitioners are more likely to be recognized and treated effectually, particularly when the specialty is so marked as it is in the branch of dental surgery. Some years ago, my father had many cases of antrum affections, mostly connected with diseased teeth, under his care; and it was the habit of that time, now some years back, for patients to bring infants to him for the sake of advice or operation during the period of dentition. But for years past the antrum cases and the babies had ceased to come under his notice, and this is the case with many affections of less importance. I cannot help thinking that dentists have to thank themselves for this partial cession of practice; for I know of instances where an opinion has been sought of the surgeon, when it was the province and duty of the dental practitioner to have acted on his own responsibility. The tendency to call in aid in comparatively simple cases is to lower the position of the dentists as a body, and to shake the confidence of the public in the estimate of their capabilities. When there is indication of disease of the maxillary bones, although such disease may be correctly diagnosed, there can be little doubt that the surgeon, from his greater experience in such cases, is the proper person to consult: but in all cases of irritative disease, caused positively or indirectly by teeth or the roots of teeth, the dentist ought to treat himself. The line between such irritative symptoms, and the appearance and conditions which present themselves to the eye and touch in malignant growths or osseous enlargements, are so marked that there is little difficulty in determining the one from the other even in the early stages. I am sure that I may say, without fear of contradiction, that cases of diseased bone or malignant growths of the investing tissues or the gums are very seldom found to have their origin in the teeth. It is true that we occasionally meet with abnormal over-development of teeth giving every appearance of diseased bone; and we not unfrequently see cases of large osseous cists connected often with the capsule of teeth, and sometimes existing *per se*. And rarely we have cases under our care of necrosis of the sockets of the teeth, the roots of which have been dead and diseased for a long while.

But time warns me that I may be taxing your listening powers and your patience beyond fair limits: as it is, I have reason to thank you for the kind attention you have bestowed during the hour devoted by custom to these preliminary emanations. In conclusion, I will urge those who are present here as pupils to continue your attendance at the hospital with the same regularity, and carry on the duties with the same zeal, which has hitherto marked the commencement of your career here. Your punctual attendance, and the evident desire to do well, evince on your parts appreciation of the opportunities afforded you of acquiring a practical knowledge of your profession. Believe me, you will find out their value when you enter into professional life; you will start at a point, to arrive at which even those who have immediately gone before you must go through an anxious time of self-taught experience.

I earnestly counsel you to look upon the calling you have selected as a profession, and to sternly uphold it as such. Make yourself acquainted with the collateral branches of science, and with general literature; thus you will gain capacity and refinement of mind which will entitle you truly to practice as, and to hold rank with, professional men, and you will add your weight in the scale to sink the folly of those who deem it politic to lower the position of the dentist, and to make him a nonentity in the professional and social scale. You will help also to shame those practitioners in medicine and surgery who appear to be so easily alarmed lest their province should be invaded by the improved education of the dentist, and who have subscribed to most illiberal and unfounded notions put forth with regard to the dental diploma issued from the College of Surgeons.

Those who, whether members of the College of Surgeons or not, devote their time and energies to the special treatment of the teeth, and the diseases consequent on their depraved condition, are not likely to practice as surgeons or medical practitioners; but they know from experience that the practice of dentistry, special as it may be, cannot be carried on with credit or efficiency without a knowledge of the principles of surgery, and an intimate acquaintance with anatomy and physiology.—*London Dental Review.*

